# ANGPTL4 overexpression inhibits tumor cell adhesion and migration and predicts favorable prognosis of triple-negative breast cancer

**DOI:** 10.1186/s12885-020-07343-w

**Published:** 2020-09-14

**Authors:** Yu-Chen Cai, Hang Yang, Ke-Feng Wang, Tan-Huan Chen, Wen-Qi Jiang, Yan-Xia Shi

**Affiliations:** 1 Sun Yat-sen University Cancer Center; State Key Laboratory of Oncology in South China; Collaborative Innovation Center for Cancer Medicine, Guangzhou, Guangdong 510060 People’s Republic of China; 2 Department of Medical Oncology, Sun Yat-sen University Cancer Center, 651 Dongfeng East Road, Guangzhou, Guangdong 510060 People’s Republic of China; 3 Department of Thoracic Surgery, The Sun Yat-sen Memorial Hospital of Sun Yat-sen University, Guangzhou, Guangdong 510120 People’s Republic of China; 4 Department of Radiation Oncology, Hui Zhou Municipal Central Hospital, Huizhou, Guangdong 516000 People’s Republic of China

**Keywords:** Triple negative breast Cancer, ANGPTL4, Prognosis, Migration, Adhesion

## Abstract

**Background:**

Triple-negative breast cancer (TNBC) patients have relatively poor clinical outcomes. A marker predicting the prognosis of patients with TNBC could help guide treatment. Extensive evidence demonstrates that angiopoietin-like 4 (ANGPTL4) is involved in the regulation of cancer growth, metastasis and angiogenesis. Therefore, its role in TNBC is of interest.

Methods: We tested the ANGPTL4 expression level in tumor tissues by immunohistochemistry (IHC) and detected its association with the clinical features of TNBC patients. Next, the effects and mechanisms of ANGPTL4 on TNBC cell migration and adhesion were investigated.

**Results:**

We found that ANGPTL4 overexpression was associated with favorable outcomes in TNBC patients. ANGPTL4 upregulation inhibited cell adhesion, migration and invasion in vitro. Further analyses demonstrated that the possible mechanism might involve suppression of TNBC progression by interacting with extracellular matrix-related genes.

**Conclusions:**

The present findings demonstrated that enhancement of ANGPTL4 expression might inversely correlate with TNBC progression. ANGPTL4 is a promising marker of TNBC and should be evaluated in further studies.

**Trial registration:**

Retrospectively registered.

## Background

Human breast cancer is the most common cancer in women worldwide and remains a global health problem [1, 2]. This disease is a heterogeneous neoplasm with various histological characteristics, molecular phenotypes, clinical characteristics and responses to therapy. Triple-negative breast cancer (TNBC) is a type of breast malignancy that is negative for the expression of estrogen receptor (ER), progesterone receptor (PR), and human epidermal growth factor 2 (HER2) [3, 4]. Patients with TNBC tends to present younger than other patients and have relatively aggressive clinical features [3, 5–7]. TNBC tumors are more sensitive to chemotherapy than other tumor types [3, 7] but cannot be treated with hormone therapies or drugs aimed at HER2; hence, there is a sharp decrease in survival compared with that of patients with hormonal receptor- or HER2-positive tumors [3]. Thus, improving the outcome of TNBC is a challenge in current clinical practice.

A previous study demonstrated that angiopoietin-like 4 (ANGPTL4) is a HIF-1 target gene that contributes to vascular infiltration [8, 9]. ANGPTL4 is a member of the angiopoietin-like protein (ANGPTL1–7) family, which has important functions in glucose and lipid metabolism [10, 11], especially as a suppressor of lipoprotein lipase (LPL) activity [12]. To date, increasingly evidences have shown that the angiopoietin family participates in the regulation of tumor growth and progression [13–15]. However, the role of ANGPTL4 expression in different kinds of malignancies appears to be different, and the precise function of this protein in cancer biology remains unclear. Recently, the ANGPTL4 protein was reported to promote or prevent tumor growth, metastasis and angiogenesis depending on the different cancer types [16]. Hence, the controversial conclusions and the possible mechanism need to be further assessed.

Importantly, chemotherapy alone is inadequate for the majority of TNBC patients. New treatment options are urgently required. In the present study, researchers detected the expression patterns of ANGPTL4 in the tumors of primary TNBC patients and investigated the effects and mechanisms of ANGPTL4 on TNBC cell migration and adhesion.

## Methods

### Patients and specimens

Patients with TNBC who were hospitalized at the Department of Medical Oncology or the Department of Breast Oncology of Sun Yat-sen University Cancer Center between January 2007 and December 2016 were retrospectively selected. The inclusion criteria were as follows: (1) patients underwent modified radical mastectomy; (2) the diagnosis of TNBC was confirmed by molecular biology and pathology; and (3) patients had complete follow-up information and pathological specimens available. The appropriate pathological samples for each patient were acquired from the Pathology Department. The final follow-up date was December 2019. Overall survival (OS) was defined as the time between diagnosis and death or the last follow-up visit. Disease-free survival (DFS) was calculated from the time of diagnosis to the date of relapse or metastasis.

### Staining and evaluation

Paraffin-embedded tissues were cut at 2 mm thickness. The slides were dewaxed and rehydrated, and endogenous peroxidase activity was blocked. The specimens were boiled in ethylenediaminetetraacetic acid (EDTA) at full power for 5 min and medium heat for 20 min for antigen retrieval. Common goat serum was used to suppress nonspecific binding. Then, polyclonal rabbit anti-ANGPTL4 antibody (diluted 1:100; Abcam, #ab115798, USA) and a secondary antibody were used. Subsequently, we applied horseradish peroxidase. Finally, hematoxylin was used to counterstain the nuclei.

All sections were separately evaluated by two independent pathologists. The percentage was determined as follows: 0–5% was scored as 0, 6–25% was scored as 1, 26–50% was scored as 2, and more than 50% was scored as 3. The intensity was calculated as follows: 0 score for no staining, 1 score for weak staining (light yellow), 2 score for yellowish brown staining, and 3 score for brown staining. The scores of proportion and intensity were added to obtain an overall score, which ranged from 0 to 6 [17]. A receiver operating characteristic (ROC) curve was applied to obtain an optimal cut-off score for overexpression of ANGPTL4 using the 0, 1 criterion.

The SPSS 22.0 statistical software package (SPSS, Inc., Chicago, IL, USA) was used to analyze all data. The variances between each groups were calculated by Student’s t-test and *p* value <0.05 was regarded as statistical significance.

### Cell culture

The breast cancer cell lines used in this research were all from the American Type Culture Collection and were identified by DNA (STR) profiling. MDA-MB-231 cells were grown in DMEM (Gibco, #41965–039) with 10% FBS (Gibco, #10270–106). BT549 cells were grown in RPMI-1640 (Gibco, # A1049101) with 10% FBS. These cell lines were cultured at 37 °C under a humidified atmosphere containing 5% CO_2_.

### Transfection and establishment of stable cell lines

The lentivirus carrying the pEZ-Lv105 plasmid encoding the full-length ANGPTL4 ORF sequence (NM_139314.3) and empty vector were purchased from GeneCopoeia. Lentivirus was transfected into cells, and puromycin (A1113803, Invitrogen; Thermo Fisher Scientific, Inc.) selection (10 μg/ml) started 24 h after transfection. Stable ANGPTL4-overexpressing MDA-MB-231 and BT-549 cells (ANGPTL4 OE) and empty vector cells (Vec) were established from isolated colonies and grown for the next assay. Untreated MDA-MB-231 and BT-549 cells were referred to as the negative control group (NC). The efficiency of ANGPTL4 gene transfection was verified by qPCR and Western blotting.

### Immunoblotting

Total cellular protein was lysed by using RIPA buffer (1:10, Cell Signaling Technology, 9806S). The BCA method (Thermo, USA) was used to calculate the protein concentrations. With a Mini Trans-Blot Electrophoretic Transfer Cell System (Bio-Rad), equal amounts of protein were subjected to gel electrophoresis. Proteins were transferred onto a PVDF membrane (Merck Millipore, Merck KGaA, Darmstadt, Germany). The membrane was blocked with 5% nonfat milk in Tris-buffered saline containing 0.1% Tween-20 for 1 h at room temperature and then incubated overnight at 4 °C with specific primary antibodies. HRP-conjugated anti-rabbit or mouse (Santa Cruz) secondary antibodies were used and visualized using a ChemiDoc imaging system.

### Wound healing assay

Cells were cultured on plastic in 6-well plates until 100% confluence, and a scratched area was created using a blue pipette tip. The gap was photographed at 0 and 16 h. The migration distance was measured in five fields randomly from every triplicate sample and is expressed as the mean versus that of the control.

### Matrigel invasion assay

A Matrigel invasion assay was performed by using Transwell polycarbonate membrane filters with a pore size of 8.0 μm (Costar, Cambridge, MA). Briefly, Matrigel was thawed, diluted and placed into the upper chamber of a 24-well Transwell. After 4 h of incubation for Matrigel gelling, the cells were harvested, resuspended in 1% FBS media at a density of 10^6^ cells/ml and placed onto the Matrigel. The lower chamber was filled with 600 μl of 10% FBS media. After a 6-h incubation at 37 °C, the nonmigrated cells on the upper surface of the membranes were eliminated by cotton swabs. The membranes were fixed with pure methanol and stained with a solution of crystal violet. Cells migrating to the substratum of the membrane were counted.

### Attachment assay

A 48-well plate was coated with 200 μl/well of 10 μg/ml fibronectin (Sigma, #F0895) in Ca and Mg-free PBS (pH 7.40) at 4 °C overnight and blocked with 200 μl of 1% heat-denatured BSA in PBS for 60 min at 37 °C. The blocking solution was discarded, and the wells were rinsed with PBS three times. Then, 100 μl of adhesion buffer was added to each well. The cells were harvested, and 50,000 cells were plated in each well and incubated for 20–30 min at 37 °C. The wells, except the standard control wells, were washed very gently 3 times until no cells were visible in the BSA-coated control wells. Then, 100 μl of adhesion buffer and 15 μl of MTT dye were added to the wells, and the plate was incubated for 4 h at 37 °C in the dark. Next, the MTT-treated cells were lysed in 100 μl of DMSO, and absorbance was measured at 570 nm on a spectrophotometer.

### Expression analysis by RNA sequencing

Total RNA from MDA-MB-231 cells with stable ANGPTL4 overexpression or the vector or NC was extracted using TRIzol (Invitrogen) according to the manufacturer’s instructions. RNA-Seq was then performed at Novogene Co., Ltd. (Beijing, P.R. China). After library preparation and cluster generation, transcriptome sequencing was performed on an Illumina HiSeq platform. Differences in the gene expression of the three groups were determined using the DESeq2 R package (1.10.1). mRNAs with an adjusted *p* value less than 0.05 detected by DESeq2 were considered differentially expressed.

### Quantitative real-time PCR

Total RNA from three groups of MDA-MB-231 cells (231 NC, 231 Vec and 231 ANGPTL4) was reverse transcribed into complementary DNA (cDNA). Then, qRT-PCR amplification was performed using FastStar Universal SYBR Green Master Mix (Roche Diagnostics GmbH, Mannheim, Germany) on an ABI 7500 Real-Time PCR Instrument (Applied Biosystems). The raw cycle number (Ct) values for each specimen were determined by the software supplied with the instrument. The relative gene expression level was computed by ΔCt (ΔCt = Ct_sample_ − Ct_ACTN_). The discrepancy between the ΔCt of the target gene and reference gene was computed by ΔΔCt (ΔΔCt = ΔCt_target gene_ – ΔCt_reference gene_). Expression fold changes were calculated by 2-ΔΔCt counts.

## Results

### The relationship between ANGPTL4 expression and the clinicopathological characteristics of TNBC patients

A total of 161 TNBC patients with a median age of 49 years (range 17–76 years) were included in this study. The median OS time was 82 months (range 0.3–153 months). Until the last follow-up investigation, 47 (29.2%) patients were identified as having recurrence or metastasis, and the disease-specific death rate was 41.8% (81/194).

**Fig. 1:**
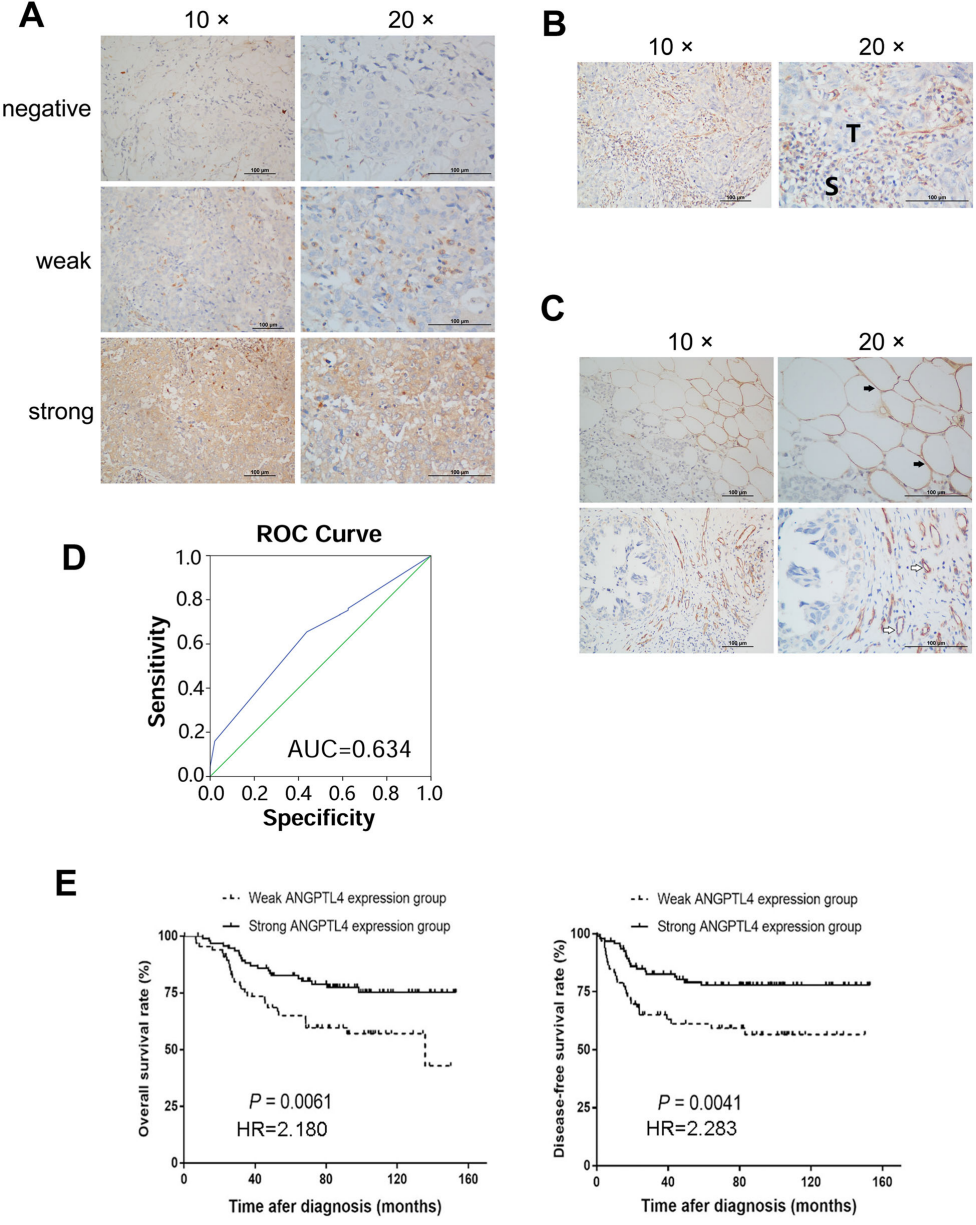
The role of ANGPTL4 expression in TNBC and its prognostic value. **a** The ANGPTL4 expression level in TNBC samples. Protein staining was mainly observed in the cytoplasm (100× and 200×). **b** Stromal cells express stronger positivity than TNBC tumor cells. **c** The expression status of ANGPTL4 in adipocytes and epithelial cells was showed, ANGPTL4 protein is overexpressed in adipocytes (solid arrow) and epithelial cells (hollow arrow). **d** The ROC curve for the scores of ANGPTL4 expression was plotted to select the appropriate cut-off score. The area under the curve was 0.634 (*p* = 0.007). **e** Kaplan–Meier survival curves of 161 patients with TNBC. The curves revealed that high ANGPTL4 expression predicted longer OS and DFS (IBM SPSS 22.0 statistical software package)

The subcellular localization and expression of the ANGPTL4 protein were observed and scored by immunohistochemistry (IHC) in various samples including 28 adjacent noncancerous tissues. According to the ROC curve results, the area under the curve was 0.634 (Fig. 1d, *p* = 0.007). An ANGPTL4 score of 4 maximized the Youden Index (sensitivity [0.655] + specificity [0.563] - 1 = 0.217) as the optimal cut-off score. Thus, patients were split into low (score < 4) and high (score ≥ 4) expression groups.

ANGPTL4 was positively expressed in the cytoplasm of TNBC cells (Fig. 1a). The high expression rate was 59% in TNBC tumor cells, which was lower than that of stromal cells (68%, Fig. 1b), especially adipocytes and epithelial cells (Fig. 1c). Weakly expressed ANGPTL4 was significantly related to the presence of neurovascular invasion (28.8% vs. 15.8%, *p* = 0.047). Additionally, a higher incidence of relapse or progression was observed in the ANGPTL4 low expression group than in the ANGPTL4 overexpression group (40.9% vs. 21.1%, *p* = 0.006). Further, the association between ANGPTL4 expression and histological differentiation was marginally significant (*p* = 0.087). However, in 161 TNBC patients, the ANGPTL4 expression level was not correlated with age, menstrual history, tumor stage or other clinical factors (Table 1).

**Table 1:** Associations between angiopoietin-like 4 (ANGPTL4) expression and clinicopathologic factors of 161 patients with TNBC

Characteristics	ANGPTL4 expression (cases)		***P*** value
	Weak	High	
**Total**	66	95	
**Age (years)**			
≤ 40	9 (13.6%)	20 (21.1%)	0.228
> 40	57 (86.4%)	75 (78.9%)	
**Family history of cancer**			0.600
No	52 (78.8%)	78 (82.1%)	
Yes	14 (21.2%)	17 (17.9%)	
**Menopause**			0.435
No	32 (51.5%)	52 (45.3%)	
Yes	34 (48.5%)	43 (54.7%)	
**History of other neoplasms**			0.601
No	63 (95.5%)	92 (96.8%)	
Yes	3 (4.5%)	3 (3.2%)	
**Stage**			0.265
0/ I/ II	44 (66.7%)	71 (74.7%)	
III	22 (33.3%)	24 (25.3%)	
**T status**			0.395
Tis/ T1/ T2	55 (83.3%)	74 (77.9%)	
T3/ T4	11 (16.7%)	21 (22.1%)	
**Lymph node metastasis**			0.139
N0	29 (43.9%)	53 (55.8%)	
N1–3	37 (56.1%)	42 (44.2%)	
**Histological differentiation**			0.087
1/ 2	24 (50.0%)	26 (38.6%)	
3	21 (50.0%)	44 (61.4%)	
**Vascular invasion**			
No	47 (71.2%)	80 (84.2%)	0.047
Yes	19 (28.8%)	15 (15.8%)	
**Disease recurrence**			
No	39 (59.1%)	75 (78.9%)	0.006
Yes	27 (40.9%)	20 (21.1%)	
**Progression**			0.333
Local recurrence	16 (59.3%)	9 (45.0%)	
Distant metastasis	11 (40.7%)	11 (55.0%)	

### Association between the expression of ANGPTL4 and TNBC prognosis

Forty-seven patients showed relapse or metastasis from the diagnosis until the final follow-up. Univariate survival analyses were performed to explore the prognostic effect of ANGPTL4 expression in TNBC patients. In the weak ANGPTL4 expression group, the median OS time was 135.7 months [95% confidence interval (CI) 55.4–216.0 months]. The 5-year OS and DFS frequencies were 64 and 56%, respectively. Nevertheless, the high ANGPTL4 expression group had a preferable prognosis: the 5-year OS and DFS rates were 82 and 77%, respectively. The Kaplan–Meier survival curves showed that ANGPTL4 overexpression indicated longer OS and DFS than low expression (both *p* < 0.05, Fig. 1e). In univariate survival analysis, patients with stage III disease, higher T stage, low ANGPTL4 expression, lymph node metastasis and vascular invasion had shorter OS and DFS times. Multivariate analysis indicated that weak ANGPTL4 expression independently predicted poor prognosis (*p* < 0.05), as did advanced disease (Tables 2 and 3).

**Table 2:** Univariate and multivariate analyses of the disease-free survival of the 161 TNBC patients

Variable		Disease-free survival		
	Univariate analysis		Multivariate analysis	
	HR (95% CI)	*P* value	HR (95% CI)	*P* value
Age ≤ 40 years old	1.090 (0.527–2.254)	0.817		
Family history of cancer	0.649 (0.290–1.448)	0.291		
Menstruating	1.321 (0.738–2.366)	0.348		
History of other neoplasms	1.321 (0.320–5.449)	0.700		
Stage III disease	3.918 (2.203–6.967)	**0.000**	2.163 (1.121–4.172)	**0.021**
T3/ T4	2.083 (1.098–3.951)	**0.025**	0.870 (0.357–2.119)	0.771
Lymph node metastasis	4.502 (2.287–8.863)	**0.000**	2.890 (1.330–6.277)	**0.007**
Poor tumor differentiation	1.426 (0.683–2.979)	0.345		
Vascular invasion	2.983 (1.639–5.429)	**0.000**	1.558 (0.731–3.320)	0.255
Low ANGPTL4 expression	2.283 (1.280–4.075)	**0.004**	2.005 (1.121–3.585)	**0.019**

Stage III disease, lymph node metastasis and low ANGPTL4 expression are independent risk factors for shorter DFS

**Table 3:** Univariate and multivariate analyses of the overall survival of the 161 TNBC patients

Variable	Overall survival			
	Univariate analysis		Multivariate analysis	
	HR (95% CI)	*P* value	HR (95% CI)	*P* value
Age ≤ 40 years old	1.133 (0.549–2.340)	0.736		
Family history of cancer	1.572 (0.705–3.505)	0.269		
Menstruating	1.131 (0.641–1.996)	0.670		
History of other neoplasms	1.232 (0.299–5.086)	0.773		
Stage III disease	4.420 (2.496–7.827)	**0.000**	2.493 (1.276–4.873)	**0.008**
T3/ T4	2.567 (1.391–4.735)	**0.003**	0.995 (0.436–2.271)	0.497
Lymph node metastasis	4.440 (2.262–8.713)	**0.000**	2.543 (1.151–5.620)	**0.021**
Poor tumor differentiation	1.547 (0.788–3.040)	0.205		
Vascular invasion	3.567 (2.002–6.355)	**0.000**	1.725 (0.841–3.535)	0.102
Distant metastasis	1.775 (0.899–3.503)	0.098		
Low ANGPTL4 expression	2.180 (1.232–3.860)	**0.006**	1.776 (0.998–3.161)	**0.051**

Stage III disease, lymph node metastasis and low ANGPTL4 expression are independent risk factors for shorter OS

### ANGPTL4 overexpression inhibits the migration and adhesion of invasive TNBC cell lines

**Fig. 2:**
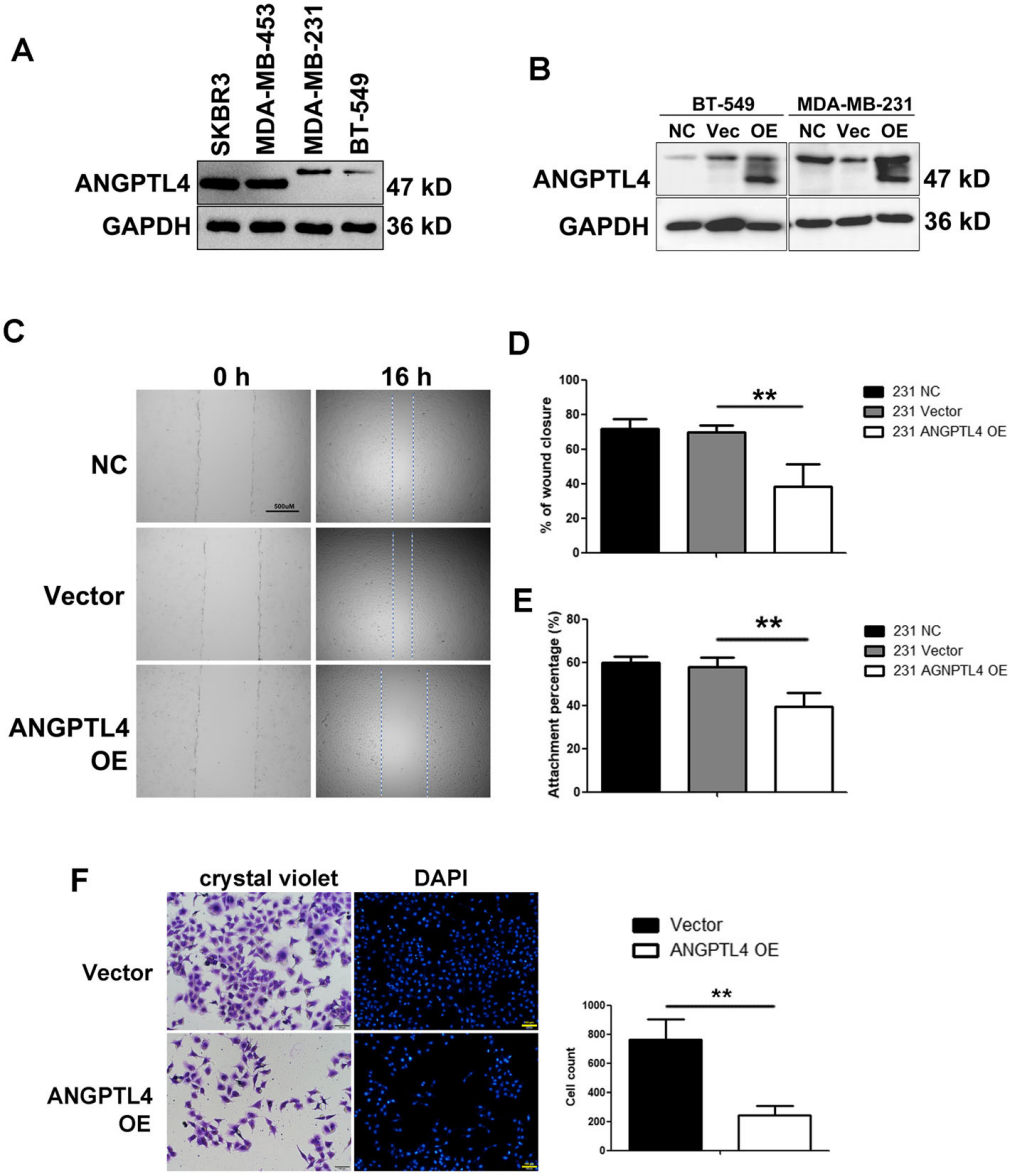
ANGPTL4 overexpression inhibits cell migration, invasion and adhesion in invasive breast cancer cell lines. **a** The expression of ANGPTL4 in luminal BC cell lines such as SKRB3 and MDA-MB-453 is higher than that in basal-like BC cell lines such as BT549 and MDA-MB-231. **b** The efficiency of transfection was confirmed by Western blots. Stable ANGPTL4-overexpressing cells (abbreviated BT549 and 231 ANGPTL4 OE) exhibited weaker wound healing (**c**), Matrigel invasion (**d**) and attachment abilities than the negative control (abbreviated NC) and empty vector cells (Graghpad Prism statistical software package, version 5.01, The Student’s t-test was used, *p < 0.05)

First, we determined the expression of ANGPTL4 in different breast cancer cell lines. In luminal BC cell lines such as SKRB3 and MDA-MB-453, the protein expression of ANGPLT4 was higher than that in basal-like BC cell lines such as BT549 and MDA-MB-231 (Fig. 2a), which are considered more invasive than luminal BC cell lines [18]. To further elucidate the role of ANGPTL4, we infected MDA-MB-231 and BT-549 cell lines with lentivirus expressing ANGPTL4, while empty vector served as the corresponding control. The efficiency of overexpression was confirmed by qPCR and Western blot analyses (Fig. 2b). To further assess the effect of ANGPTL4 in TNBC, we detected the impact of ANGPTL4 overexpression on cell migration, invasion and adhesion. In an in vitro scratch-wound assay (Fig. 2c), 16 h after scratching, the percentage of wound closure in the 231 vector group was 69.9 ± 4.0%, while that in the 231 ANG OE group was 38.5 ± 12.7% (*p* < 0.01). The percentage of wound closure in the 549 vector group was 61.9 ± 5.0%, while that in the BT-549 ANG OE group was 43.9 ± 9.8% (*p* < 0.05). In the Matrigel invasion assay, the invaded cell number of the 231 vector group was 761 ± 142, while that of the 231 ANG OE group was 245 ± 63 (Fig. 2d, *p* < 0.05). The results from the wound healing assay and Matrigel invasion assay showed that ANGPTL4 overexpression evidently attenuated the cell invasive and migratory abilities compared with the control. In the cell adhesion assay, the attachment percentage of the 231 vector group was 57.7 ± 4.5%, while that of the 231 ANG OE group was 39.4 ± 6.6% (Fig. 2e, *p* < 0.05), suggesting that ANGPTL4 overexpression inhibits cell adhesion and attachment, preventing cell migration and invasion.

### ANGPTL4 overexpression decreases the mRNA levels of extracellular matrix (ECM)-related genes

**Fig. 3:**
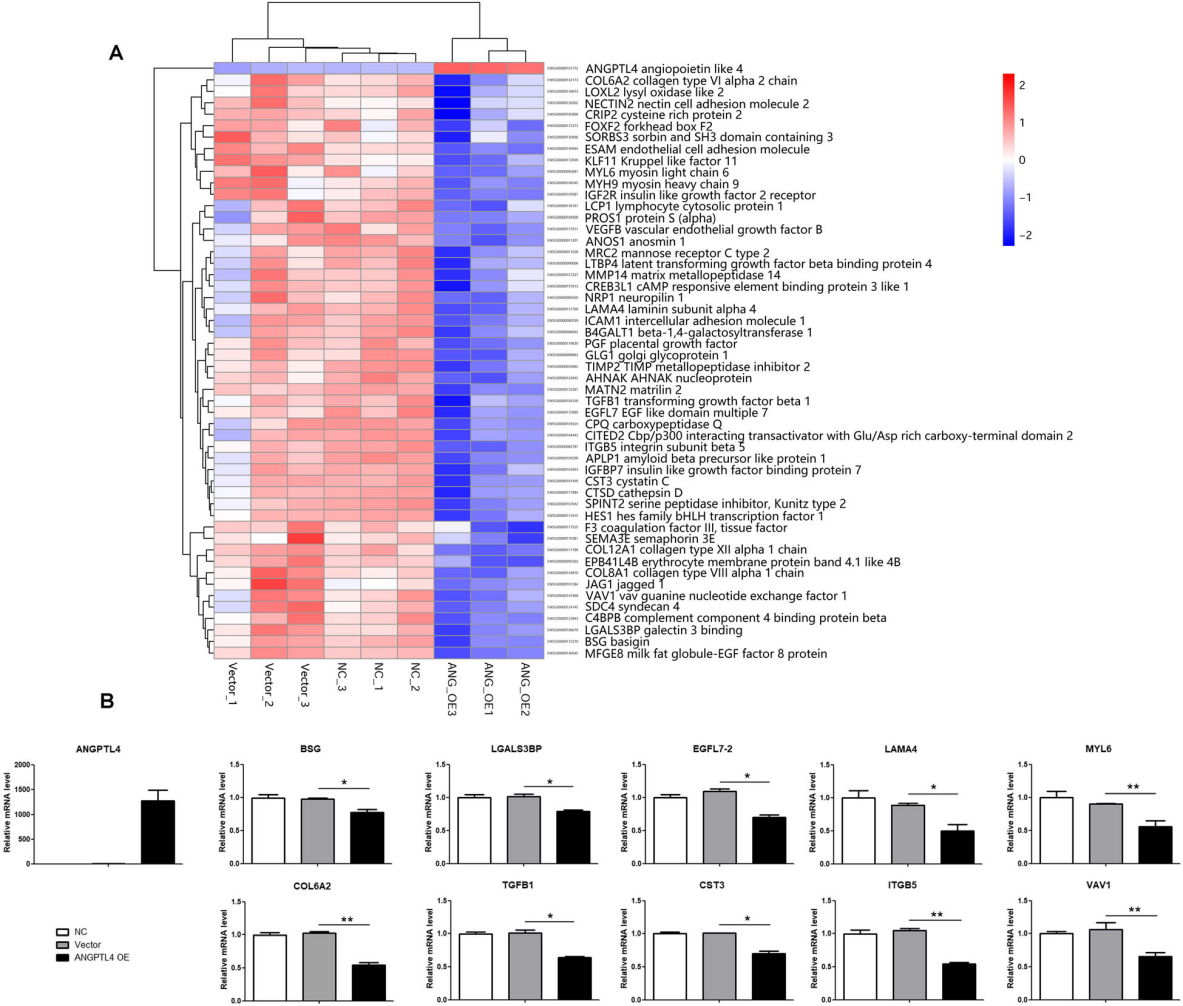
ANGPTL4 overexpression decreases the mRNA levels of ECM-related genes. **a** Next-generation RNA sequencing revealed that genes related to adherens junctions, blood vessel morphogenesis, extracellular matrix and wound healing were most affected by ANGPTL4 overexpression. **b** Real-time quantitative PCR confirmed that ten genes were downregulated in the 231 ANGPTL4 OE group compared with the control group. These results suggest that ANGPTL4 overexpression might affect TNBC progression by inhibiting ECM-related genes (Graghpad Prism statistical software package, version 5.01, *p < 0.05)

To further understand how ANGPTL4 inhibits tumor migration, we performed next-generation RNA sequencing on three groups of MDA-MB-231 cell lines: the NC (231 NC), vector (231 vector) and ANGPTL-overexpressing groups (231 ANGPTL4 OE). Differentially expressed gene (DEG) analysis and GO (Gene ontology, http://www.geneontology.org/) enrichment revealed that genes included in the pathways of adherens junction, blood vessel morphogenesis, extracellular matrix and wound healing were most affected by ANGPTL4 overexpression (Fig. 3a). Real-time quantitative PCR was performed to confirm the results of RNA sequencing. Among 52 genes, ten were significantly downregulated in the 231 ANGPTL4 group compared with the 231 vector group, which was consistent with the RNA sequencing results (Fig. 3b). These genes are *BSG* (basigin), *LGALS3BP* (galectin 3 binding), *EGFL7–2* (EGF-like domain multiple 7), *LAMA4* (laminin subunit alpha 4), *MYL6* (myosin light chain 6), *COL6A2* (collagen type VI alpha 2 chain), *ITGB5* (integrin subunit beta 5), *TGFB1* (transforming growth factor beta 1), *CST3* (cystatin C), and *VAV1* (vav guanine nucleotide exchange factor 1).

## Discussion

ANGPTL4 is secreted by cells that can cleave it into two subtypes. Native full-length ANGPTL4 (flANGPTL4) can produce the COOH-terminal fibrinogen-like fragment (cANGPTL4) and the N-terminal coiled-coil domain (nANGPTL4) via proteolytic processing [19]. To determine whether ANGPTL4 overexpression correlates with the prognosis of TNBC, we analyzed the level of flANGPTL4 in cancer cells by IHC. We confirmed that high flANGPTL4 expression was associated with lower relapse and vascular invasion rates than weak expression. Next, we observed that flANGPTL4 overexpression inhibits cell adhesion and attachment, which leads to inhibition of cell invasion and migration. Various studies have also shown that ANGPTL4 expression prevents metastasis and angiogenesis by reducing vascular leakiness, cell motility and invasiveness in different neoplasm types, including melanoma, gastric, lung and colorectal tumors, as well as metastases [20–22]. Strong ANGPTL4 expression in nude mouse xenografts also inhibited metastasis through suppression of tumor cell migration and invasiveness [21]. In the present study, we demonstrated that ANGPTL4 overexpression decreased the vascular invasion and relapse rate in patients, which are factors related to aggressiveness and invasion. These results were consistent with the phenomena observed in vitro.

However, many studies have reported that ANGPTL4 expression increases cancer cell aggressiveness and migration [9, 23–27]. For instance, Kim and his colleagues demonstrated that ANGPTL4 induction by hypoxia facilitated the growth of colorectal cancer [25], and Li et al. announced that HIF-1α-activated ANGPTL4 overexpression contributes to tumor metastasis in hepatocellular carcinoma (HCC) [9]. Notably, previous studies have shown conflicting results in breast cancer research. One team discovered that the expression of ANGPTL4 could be induced by TGFβ, which could facilitate lung metastasis [23]. Others have shown that adipocyte-derived ANGPTL4 drives disease progression under obese conditions [27]. Zhang et al. revealed that the downregulation of HIF-1α expression in breast cancer cells suppressed primary tumor progression and inhibited the metastasis of tumor cells to the lungs by reducing ANGPTL4 expression [8]. In addition, previous studies showed that the copy number of ANGPTL4 increased in the circulating tumor cells of patients and was related to increased aggressiveness in breast cancer [28]. Additionally, ANGPTL4 overexpression was related to a short DFS in a basal breast cancer type commonly found in young women [29].

These conflicting phenomena might be given rise to the following possibilities: First, different ANGPTL4 fragments may have distinct biological roles in human cancers. Native flANGPTL4 can suppress tubule formation and endothelial cell migration [22]. nANGPTL4 binds to LPLs to inhibit their activities [12, 16]. cANGPTL4 may have various effects, including regulating cancer progression [16, 30, 31]. The investigators of the present study demonstrated that flANGPTL4, and not nANGPTL4 or cANGPTL4, was responsible for inhibition of TNBC progression in vitro and favorable prognosis in vivo. Second, despite discrepancies in these results, early studies have used various cancer cell samples, suggesting that the disparate influences of ANGPTL4 in cancer progression may be determined by cancer types. Third, the primary source and tumor microenvironment of ANGPTL4 may affect biological behaviors. For example, Ryan Kolb et al. hypothesized that the main source of cANGPTL4 could be adipose cells in the breast cancer microenvironment. Moreover, a reduction in cancer progression was observed when ANGPTL4 was maintained in the tumor cells but reduced in the microenvironment [27]. Another study found that circulating ANGPTL4 in the tumor microenvironment might be excreted by other cell types, such as adipocytes, which could accelerate cell proliferation and metastasis [31]. Although ANGPTL4 was highly expressed in adipocytes and epithelial cells, we only focused on the role of flANGPTL4 from cancer cells, not stromal cells. These hypotheses suggest that the expression level and effects of ANGPTL4 in cancer may be context- and tumor-type-dependent, which may explain the diversity of previous studies.

We further explored the potential mechanisms by which ANGPTL4 regulates TNBC progression, and we performed next-generation RNA sequencing to identify the receptors of ANGPTL4. The results revealed that genes included in the ECM were most affected by ANGPTL4 overexpression. Notably, ANGPTL4 is a specific matricellular protein that is considered to interact with specific integrins and ECM proteins to affect cell migration [32, 33]. The downstream receptors that regulate the functions of ANGPTL4 are still unclear. A study showed that the tumor-facilitating effect of ANGPTL4 is strongly associated with PGE2 and hypoxia [25]. Additionally, ANGPTL4 is believed to interact with other molecules such as reactive oxygen species (ROS) to regulate anoikis resistance and antiapoptotic effects [25, 34, 35]. A study found that modification of ANGPTL4 might inhibit the MEK/ERK pathway in endotheliocytes, suppressing angiogenesis induced by VEGF [36]. Moreover, the VCAM-1/integrin b1 [9] and Rac/PAK signaling pathways [24] were activated by increasing the ANGPTL4 interaction with specific factors. Based on these and our results, it is likely that ANGPTL4 inhibits TNBC adhesion and migration via ECM-related biological signals. Thus, further studies are needed to illuminate the potential mechanisms by which ANGPTL4 regulates cancer development.

## Conclusions

In summary, our findings demonstrate that the overexpression of flANGPTL4 in cancer cells is strongly associated with favorable clinical outcomes in TNBC patients. Furthermore, our initial results suggest that overexpression of ANGPTL4 in TNBC cells may inhibit cell adhesion and attachment. In addition, the upregulation of ANGPTL4 reduces the mRNA levels of ECM-related genes, indicating that ANGPTL4 contributes to TNBC progression by suppressing ECM-related proteins. Hence, we believe ANGPTL4 is a potential prognostic marker and therapeutic target for TNBC patients.

## Supplementary information

**Additional file 1.** The gels are represented for ANGPTL4 in Figure2A. The gels are represented for GAPDH in Figure2A. The gels are represented for ANGPTL4 in Figure2B. The gels are represented for GAPDH in Figure2B.

## Data Availability

The datasets used and analyzed during the current study are available from the corresponding author on reasonable request.

## Abbreviations

TNBC: Triple-negative breast cancer
ANGPTL4: Angiopoietin-like 4
IHC: Immunohistochemistry
ER: Strogen receptor
PR: Progesterone receptor
HER2: Human epidermal growth factor 2
LPL: Lipoprotein lipase
OS: Overall survival
DFS: Disease-free survival
EDTA: Ethylenediaminetetraacetic acid
ROC: Receiver operating characteristic
HR: Hazard ratio
CI: Confidence interval
