# Supplementary material for: ANGPTL4 overexpression inhibits tumor cell adhesion and migration and predicts favorable prognosis of triple-negative breast cancer
Source: BMC Cancer. 2020 Sep 14;20:878. doi: 10.1186/s12885-020-07343-w (PMC7489026; doi:10.1186/s12885-020-07343-w)

Additional Figure 1. The gels are represented for ANGPTL4 in Figure2A


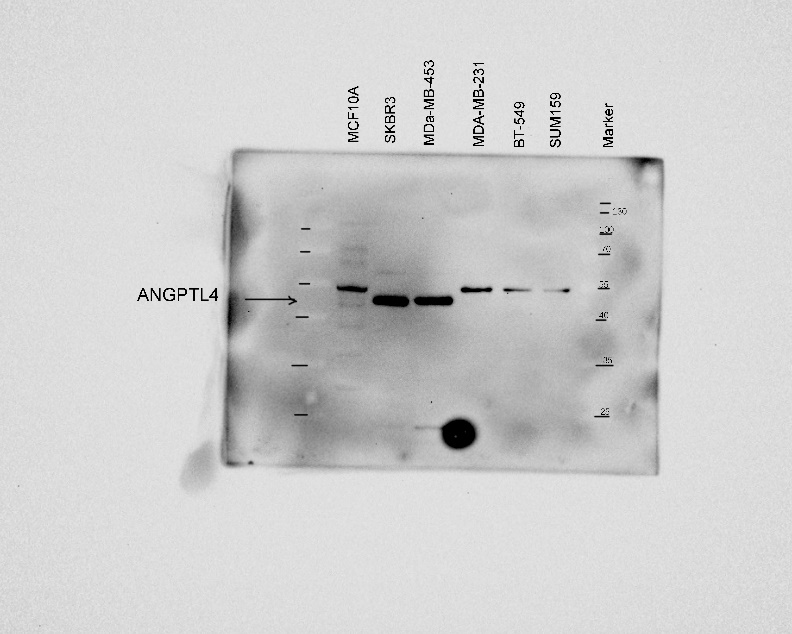


Additional Figure 2. The gels are represented for GAPDH in Figure2A


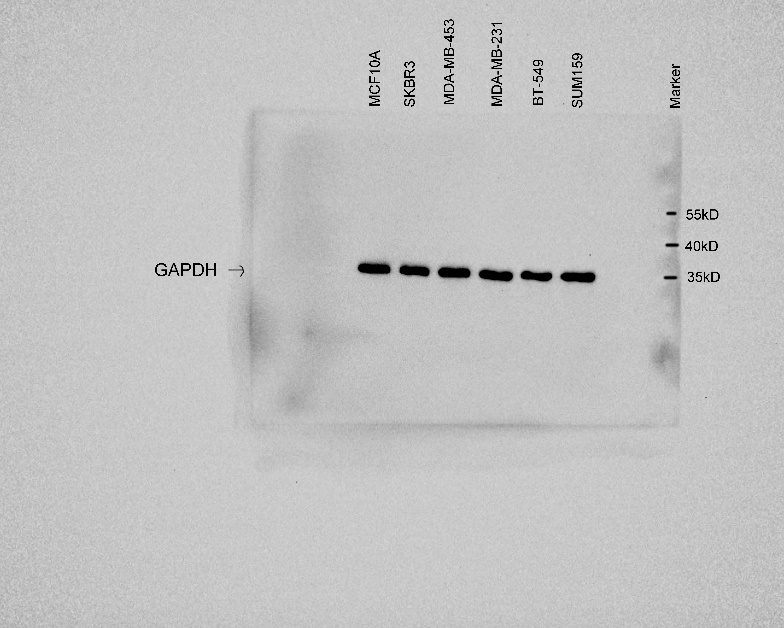


Additional Figure 3. The gels are represented for ANGPTL4 in Figure2B


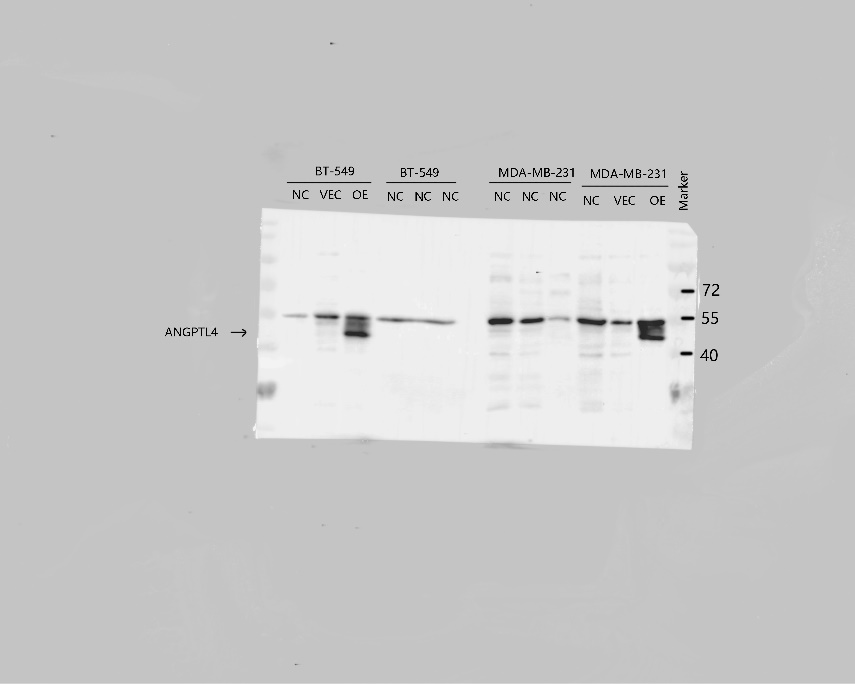


Additional Figure 4. The gels are represented for GAPDH in Figure2B


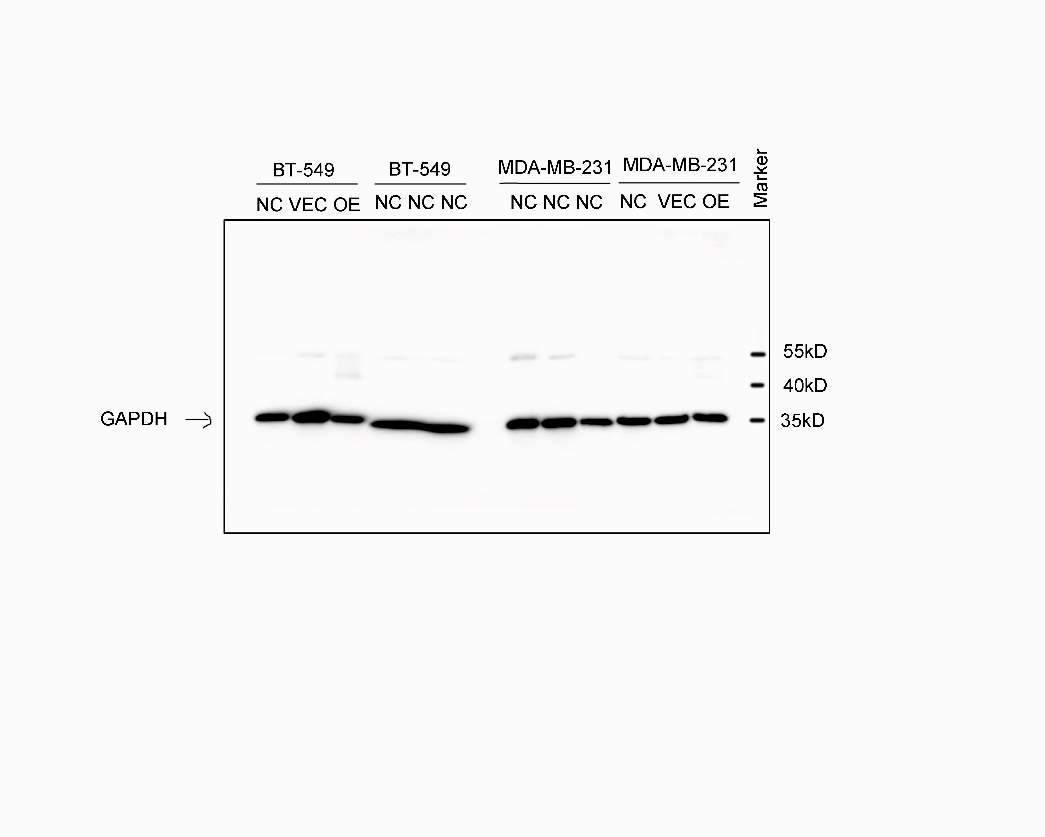

Supplement: Supplementary file 1 — Additional file 1. The gels are represented for ANGPTL4 in Figure2A. The gels are represented for GAPDH in Figure2A. The gels are represented for ANGPTL4 in Figure2B. The gels are represented for GAPDH in Figure2B. [file 12885_2020_7343_MOESM1_ESM.docx]
